# Site-Specific Labeling of Protein Kinase CK2: Combining Surface Display and Click Chemistry for Drug Discovery Applications †

**DOI:** 10.3390/ph9030036

**Published:** 2016-06-27

**Authors:** Christian Nienberg, Anika Retterath, Kira-Sophie Becher, Thorsten Saenger, Henning D. Mootz, Joachim Jose

**Affiliations:** 1 Institut für Pharmazeutische und Medizinische Chemie, PharmaCampus, Westfälische Wilhelms-Universität Münster, Corrensstraße 48, D-48149 Münster, Germany; christian.nienberg@uni-muenster.de (C.N.); a_rett04@uni-muenster.de (A.R.); thorsten.saenger@uni-muenster.de (T.S.); 2 Institut für Biochemie, Westfälische Wilhelms-Universität Münster, Wilhelm-Klemm-Straße 2, D-48149 Münster, Germany; kira.becher@uni-muenster.de (K.-S.B.); henning.mootz@uni-muenster.de (H.D.M.)

**Keywords:** CK2, kinase, Autodisplay, click chemistry, unnatural amino acid, bioorthogonal, labeling, drug discovery, protein-protein interaction

## Abstract

Human CK2 is a heterotetrameric constitutively active serine/threonine protein kinase and is an emerging target in current anti-cancer drug discovery. The kinase is composed of two catalytic CK2α subunits and two regulatory CK2β subunits. In order to establish an assay to identify protein-protein-interaction inhibitors (PPI) of the CK2α/CK2β interface, a bioorthogonal click reaction was used to modify the protein kinase α-subunit with a fluorophore. By expanding the genetic code, the unnatural amino acid para azidophenylalanine (pAzF) could be incorporated into CK2α. Performing the SPAAC click reaction (Strain-Promoted Azide-Alkyne Cycloaddition) by the use of a dibenzylcyclooctyne-fluorophore (DBCO-fluorophore) led to a specifically labeled human protein kinase CK2α. This site-specific labeling does not impair the phosphorylation activity of CK2, which was evaluated by capillary electrophoresis. Furthermore a dissociation constant (*K_D_*) of 631 ± 86.2 nM was determined for the substrate α_S1_-casein towards CK2α. This labeling strategy was also applied to CK2β subunit on *Escherichia coli*, indicating the site-specific modifications of proteins on the bacterial cell surface when displayed by Autodisplay.

## 1. Introduction

Human protein kinase CK2 was discovered in 1954 by Burnett and Kennedy [1] and is a constitutively active serine/threonine kinase. Erroneously the kinase was first named casein kinase 2 persuading caseins as in vivo substrates. Today’s literature postulates that caseins are only in vitro substrates of CK2 [2]. The CK2 holoenzyme forms a heterotetrameric structure composed of two catalytically active α- and two regulatory β-subunits, which are dimerized by a zinc finger [3]. The α-subunit can be replaced in some cases by the isoform CK2α’ [4]. CK2 is a highly pleiotropic protein kinase, phosphorylating a huge number of cellular substrates [5] and is involved in many cellular processes [6]. The kinase is related to a variety of human diseases and represents an important target in current cancer research [7,8]. Currently, plenty of inhibitors are known to inhibit the phosphorylation activity of CK2 including dibenzo[*b*,*d*]furane- [9] and indeno[1,2-*b*]indole-derivatives [10]. One of the most potent ATP-competitive inhibitors found so far is CX4945, which currently is in clinical trials for approval as anti-cancer agent [11]. Beside inhibitors binding to the ATP binding pocket, some compounds interfere with the interaction of the CK2α- and the CK2β-subunit. The cyclic peptide Pc, derived from the C-terminal CK2β segment, is an effective CK2β-competitive compound [12]. Central points for the identification and characterization of new inhibitors or interaction partners of CK2 are screening- and protein-protein interaction assays, which often require the modification by a fluorophore of the target enzyme CK2. Methods including flow cytometry, microscale thermophoresis (MST), FRET- or anisotropy measurements for these tests, are based on the detection of a fluorescently labeled protein [12,13,14,15]. Most commercially available labeling applications attack lysine and cysteine side chains of proteins. These procedures can lead to modifications at different positions and different protein-to-fluorophore ratios, which can result in heterogeneously labeled products. The consequences can be altered affinities, stabilities and potential changes in protein activity in contrast to the unlabeled protein. Modifying proteins only in one well-selected position could yield a specific labeling without any influence on protein folding along with activity and results in a homogenously labeled protein solution. The advantage of a specific modification by incorporating an unnatural amino acid with appropriate functional groups, has already been shown for antibody-drug conjugates with regard to selectivity and potency [16]. Unnatural amino acids facilitate bioorthogonal reactions and expand the capabilities of protein chemistry. Among others, these comprise the creation of cyclic peptides by an incorporation of an unnatural amino acid followed by an oxime ligation [17] as well as site-specific chemical-tag labeling of proteins by recombinant split inteins [18].

The Autodisplay technology is based on the natural secretion mechanism of autotransporter proteins in gram-negative bacteria. For cathepsin G, a target in chronic inflammatory diseases such as lung emphysema, new peptidic inhibitors were identified by using the binding affinity of the fluorescent labeled target protein to a surface translocated peptide library on *Escherichia coli* [19]. The heat shock protein HSP90, a homodimer, was also combined with the secretion mechanism of Autodisplay and enabled the identification of peptides, which inhibited the dimerization of HSP90 [20]. In previous studies the successful display of the heterotetrameric CK2 holoenzyme on the surface of *E. coli* was reported [21]. Recently the Autodisplay of CK2α′ was shown and enabled inhibitor testing by capillary electrophoresis of the less investigated isoform of CK2α [22]. Combining a specifically labeled protein with the Autodisplay mediated surface display enables a variety of possibilities for new applications based on fluorescence detection.

In this study, a specific labeling of the human protein kinase CK2α subunit and surface translocated CK2β-subunit on *E. coli* cells generated by an incorporation of the unnatural amino acid pAzF followed by a bioorthogonal click reaction is reported. The advantages of a specific protein modification as well as advantages for drug discovery, using microscale thermophoresis (MST), with the target enzyme CK2α were confirmed.

## 2. Results and Discussion

### 2.1. Selecting a Suitable Position in CK2α for a Specific Fluorophore Labeling

Protein labeling of the target CK2 is an important basis for several methods based on fluorescence detection with the aim to discover and investigate inhibitors or binding partners. Performing a labeling reaction of CK2 by fluorescein isothiocyanate (FITC), which is reactive towards nucleophiles including amine sidechains, revealed a loss of phosphorylation activity in this study.

**Figure 1:**
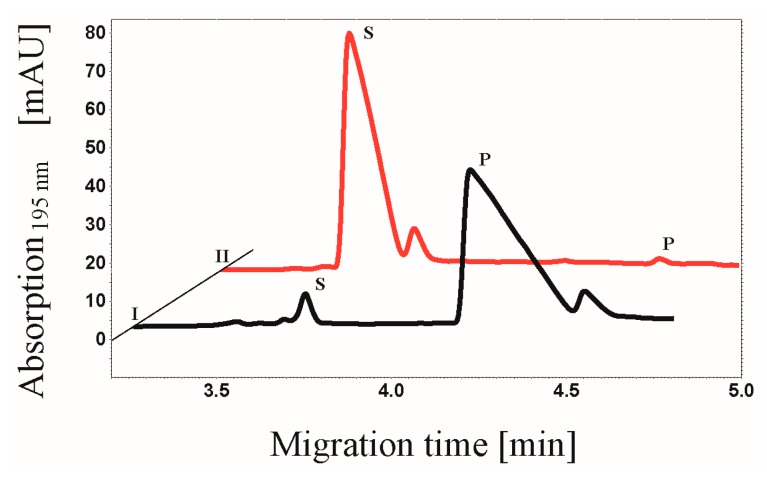
Comparison of the phosphorylation activity of the heterotetrameric CK2 before and after reaction with FITC. The CE-based assay as described before by Gratz et al. [23] was used to determine the CK2 activity. Electropherogram of the phosphorylation of the substrate peptide RRRDDDSDDD (114 µM) by unlabeled (I, 2.6 µg) and fluorescein-conjugated CK2 (II, 2.6 µg) after an incubation time of 30 min is shown. Substrate (S) and product (P) peaks were detected after 3.7 min and 4.3 min, respectively.

The kinase activity of CK2 on the substrate peptide RRRDDDSDDD was determined by a capillary electrophoresis assay [23], which is based on a different migration time of the phosphorylated product in contrast to the unphosphorylated substrate through a difference in charge. Three independent batches of labeled CK2-FITC were investigated and exhibited slight or no phosphorylation activity. A typical activity measurement as obtained with one of these batches indicating a minimal phosphorylation activity of CK2-FITC in comparison to the unlabeled CK2 after 30 min of incubation time with the substrate peptide is shown in Figure 1. These results led to the conclusion that unspecific protein modifications as obtained with FITC have a negative influence on CK2 activity. CK2α contains 23 lysines. Modifications of lysine residues in the sequence of CK2α by FITC could have resulted in heterogeneously labeled products as well as in differences in the CK2α to fluorophore ratio. A coupling of FITC to K68, which is located at the ATP binding site [24], could for example interfere with the binding of the co-factor ATP in CK2α subunit and hence the loss of enzymatic activity. In addition, a labeling reaction of K191 of the regulatory CK2β dimer by FITC could have hindered the interaction with the CK2α subunit and hence lead to a reduction of enzymatic activity.

A specific labeling of the enzyme at a distinct position could overcome these effects. The method of Chin et al. [25] enables a site-specific incorporation of the unnatural amino acid para azidophenylalanine (pAzF) into proteins.

**Figure 2:**
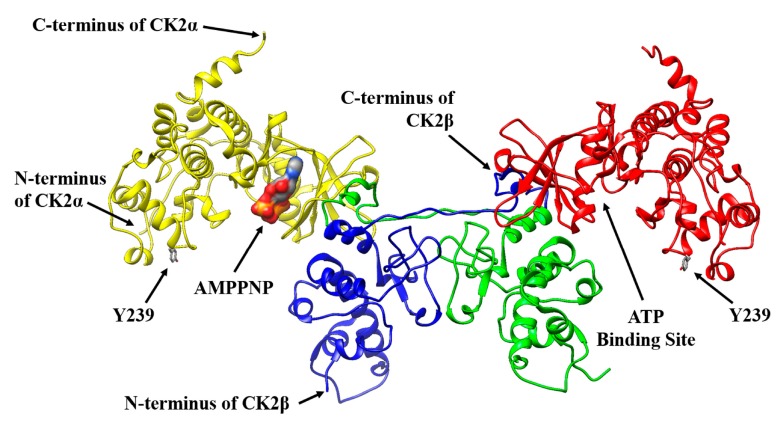
Ribbon diagram illustrating the structure of heterotetrameric human protein kinase CK2. For this purpose CK2 structure (PDB identification number 1JWH) was processed with the UCSF Chimera 1.10.2 software package [26]. The catalytic CK2α subunit binds to the regulatory CK2β subunit. Dimerization of two β-subunits is mediated by a zinc finger. The non-hydrolysable ATP analogue adenosine 5´-[β,γ-imido]triphosphate (AMPPNP) is bound in the ATP binding pocket of one catalytic α-subunit. A tyrosine in position 239 (Y239) was chosen to be replaced by the unnatural amino acid pAzF into CK2α.

The incorporation of pAzF into CK2α, which can easily be modified with a fluorophore by click reaction, could avoid a negative effect on the phosphorylation activity of human protein kinase CK2. For the incorporation of the unnatural amino acid, tyrosine Y239 in the sequence of CK2α was chosen. This position shows a sufficient distance to the ATP binding site and to the interaction site with the CK2β subunit. In addition, a tyrosine as chosen for substitution has structural similarity to pAzF and it is located at the periphery of the α-subunit structure (Figure 2) and hence supposed to have minimal effects on the correct folding of the protein.

### 2.2. Incorporation of pAzF into CK2α

The unnatural amino acid pAzF could be incorporated in the CK2α-subunit in *E. coli* by the use of an orthogonal amber suppressor tRNA, which incorporates the unnatural amino acid at an amber stop codon (UAG) [27]. Therefore, site-directed mutagenesis was applied to modify the gene encoding CK2α, resulting in the replacement of the codon for Y239 (TAT) by the amber stop codon TAG. The corresponding plasmid was termed pCK2α^Y239Stop^. The biosynthesis of the mutated CK2α-pAzF was controlled by the T7-promotor. *E. coli* BL21(DE3) cells were transformed with the plasmid pCK2α^Y239Stop^ and a second plasmid called pEVOL-pAzF, directing the expression of the genes for the amber suppressor tRNA and an aminoacyl-tRNA synthetase [25]. The aminoacyl-tRNA synthetase acylates the tRNA with the unnatural amino acid pAzF, in case it is supplied to the growth medium. The amber tRNA recognizes the amber stop codon UAG, followed by the incorporation of pAzF into the CK2α amino acid sequence. Expression of the amber tRNA was under control of a constitutive promotor. Two similar genes of the aminoacyl-tRNA synthetase are encoded by the pEVOL plasmid, which resulted in higher yields of the mutated proteins as described before by Young et al. [28]. Expression of one of the aminoacyl-tRNA synthetases was under control of a constitutive promoter, whereas expression of the other was inducible by arabinose. To maintain both plasmids in one cell of *E. coli*, they were equipped with two different origins of replication, ColE1 for pCK2α^Y239Stop^ and p15A for pEVOL-pAzF. In addition pCK2α^Y239Stop^ encoded a carbenicillin resistance, whereas pEVOL-pAzF encoded a chloramphenicol resistance. Only if both plasmids were present, translation of the amber stop codon UAG would be possible, resulting in the incorporation of the unnatural amino acid pAzF into the amino acid sequence of CK2α.

**Figure 3:**
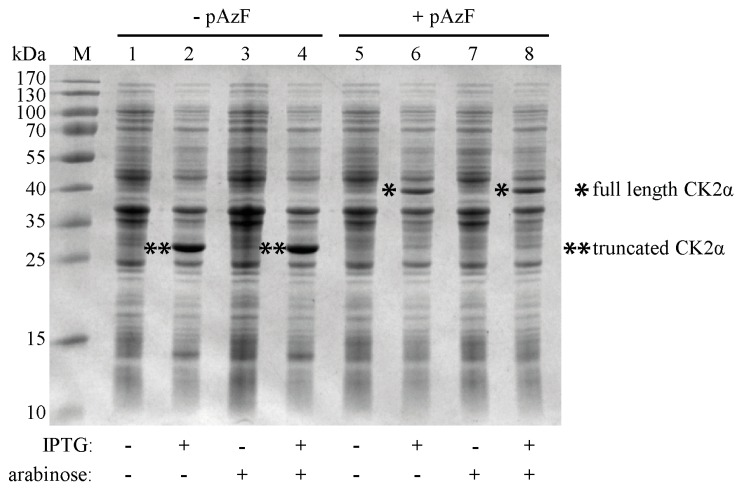
SDS-PAGE analysis of gene expression and incorporation of pAzF into CK2α. The addition or the omission of the unnatural amino acid pAzF, the inducers IPTG and arabinose to *E. coli* BL21(DE3) cells with the plasmids CK2α^Y239Stop^ and pEVOL-pAzF, expressing the mutated CK2α (IPTG) and the amber suppressor tRNA (constitutive)/aminoacyl-tRNA synthetase (constitutive/arabinose), were proven in a volume of 1 mL minimal medium for each case. Cells were boiled for 20 min at 95 °C and protein lysates were separated on 10% acrylamide. The apparent molecular mass of the marker proteins is shown in lane M. Full-length CK2α (40 kDa) could be synthesized in lane 6 and 8, i.e., when all components were present. Because of the stop codon UAG and the lack of pAzF, the truncated CK2α (28 kDa) appeared in lane 2 and 4.

First, the influence of the expression-inducing agents isopropyl-β-d-thiogalactopyranoside (IPTG) and arabinose on the gene expression of both plasmids, pCK2α^Y239Stop^ and pEVOL-pAzF, was investigated together with the effect of presence and absence of the unnatural amino acid pAzF in the growth medium. For each combination the bacterial cells were boiled and the proteins were separated by SDS-PAGE (Figure 3). In the presence of the inducer IPTG and in absence of the unnatural amino acid pAzF a truncated CK2α-isoform with a molecular weight of 28 kDa appeared, because the aminoacyl-tRNA synthetase could not acylate the orthogonal tRNA with pAzF. As a consequence the protein translation was terminated at the amber stop codon UAG. The lack of full length CK2α-pAzF also indicates that there was no readthrough across the amber stop codon in the sequence of pCK2α^Y239Stop^ [29]. Full-length CK2α with a molecular weight of 40 kDa could only be detected when both inducers, IPTG as well as arabinose and in addition the unnatural amino acid pAzF were present (Figure 3, lane 8). The addition of the inducer arabinose was not essential for the biosynthesis of full-length CK2α, because of the second copy of the aminoacyl-tRNA synthetase controlled by the constitutive promotor on the pEVOL plasmid (Figure 3, lane 6). These results indicate that both plasmids were present and that the unnatural amino acid pAzF was successfully incorporated in the catalytic subunit CK2α in case it was present in the growth medium.

### 2.3. Purification and Click Chemistry of CK2α-pAzF

It was intended to use a bioorthogonal click reaction to modify purified CK2α-pAzF with a fluorophore and to confirm the accessibility of the azido group of the incorporated pAzF. In order to obtain CK2α-pAzF in larger amounts, bacterial cells were grown in 1.2 L minimal medium to the mid log phase. The unnatural amino acid pAzF (1 mM) was added to the cell suspension and gene expression was induced by the addition of IPTG (1 mM) and arabinose (0.2%) for 4 h at 30 °C. After cultivation, *E.* *coli* cells were harvested and disrupted by sonication. Subsequently the cell lysate was centrifuged and CK2α-pAzF was purified by P11 phosphocellulose chromatography according to Grankowski et al. [30]. A linear gradient of 300 mM to 1500 mM NaCl was used to eluate bound proteins. Fractions of CK2α-pAzF were received at a concentration of approximately 600–700 mM NaCl and analyzed by SDS-PAGE. Finally, CK2α-pAzF was obtained in a concentration of approximately 130 µg/mL and a total yield of 2.7 mg. CK2α-pAzF was concentrated by ultrafiltration to a final concentration of 1.1 mg/mL.

**Figure 4:**
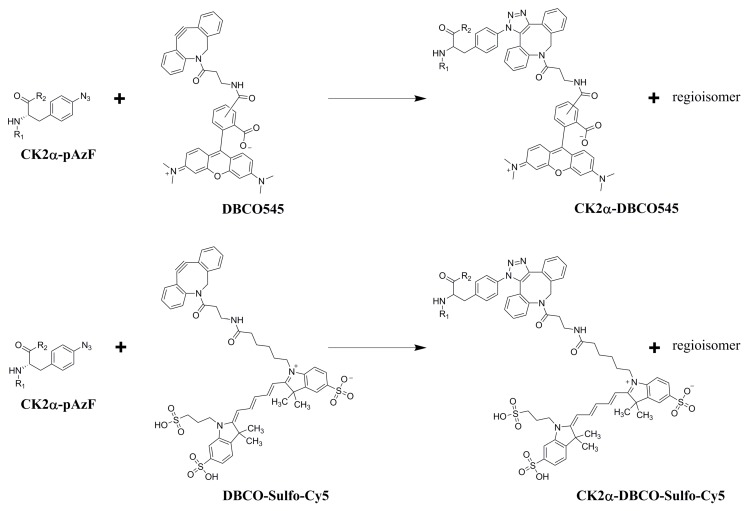
SPAAC click reaction of CK2α-pAzF with the two dibenzylcyclooctyne-fluorophores DBCO545 and DBCO-Sulfo -Cy5, respectively (R_1_ = N-terminal sequence of CK2α-pAzF, R_2_ = C-terminal sequence of CK2α-pAzF). For both cases, beside the regioisomer 1,4 as shown in the reaction scheme, the regioisomer 1,5 can be obtained as well.

For the confirmation of the successful incorporation of pAzF, a Strain Promoted Azide-Alkyne Cycloaddition (SPAAC) reaction was performed with a dibenzylcyclooctyne-fluorophore [31]. This click reaction is only feasible in the presence of pAzF in the amino acid sequence of CK2α. The functional azido group of the incorporated unnatural amino acid pAzF reacts in a 1,3-dipolar cycloaddition with the DBCO-fluorophore to the specifically labeled CK2α. In this study two different fluorophores were used for the click reaction, dibenzylcyclooctyne-fluor 545 (DBCO545) and dibenzylcyclooctyne-Sulfo -Cy5 (DBCO-Sulfo -Cy5) (Figure 4). The SPAAC reaction between the purified CK2α-pAzF (130 µg/mL in buffer P50) and the respective DBCO-fluorophore (50 µM) was performed for 1 h in the dark at room temperature (RT). When using SDS-PAGE, CE-measurements or flow cytometry, the DBCO-fluorophore coupled CK2α was directly applied. For MST measurements, an additional ultrafiltration step (vivaspin500 columns, Sartorius, Göttingen, Germany) was performed in order to remove the unbound fluorophore.

**Figure 5:**
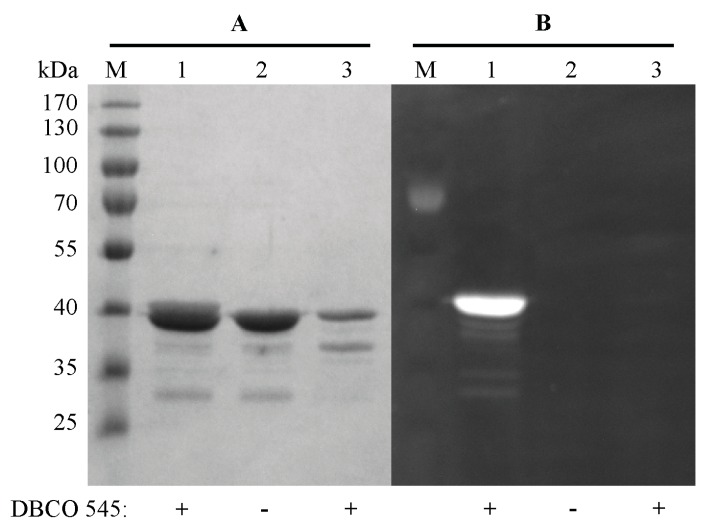
SDS-PAGE analysis of the SPAAC click reaction between CK2α-pAzF and the fluorophore DBCO545. Protein solutions were separated on 10% acrylamide. In lane M, the apparent molecular mass of the marker proteins is given. Purified and concentrated CK2α-pAzF (11 µg) in presence and absence of DBCO545 in a final concentration of 50 µM are shown in lane 2 and lane 1, respectively. As control purified full length CK2α (2.8 µg) without incorporated pAzF was also incubated with DBCO545 (lane 3). (**A**) Proteins were stained with Coomassie brilliant blue G250. (**B**) Visualization of the fluorescent protein band of CK2α-DBCO545 by LED-illuminator (470 nM).

The resulting CK2α-DBCO545 was analyzed by gel electrophoresis. As a control purified full length CK2α without mutation and hence also without incorporated pAzF, which was incubated with DBCO545 as well, was analyzed in comparison. The protein band of CK2α-DBCO545 could be visualized by a LED-illuminator (470 nm) and showed the expected fluorescence intensity (Figure 5B). Lacking fluorescence of the control CK2α without incorporated pAzF confirmed the bioorthogonal SPAAC click reaction. The gel was also stained with Coomassie brilliant blue G250 for visualization of all protein bands (Figure 5A).

### 2.4. Proof of Phosphorylation Activity of CK2α-pAzF/CK2α-DBCO545

The purified CK2α-pAzF was tested on phosphorylation activity towards the substrate peptide RRRDDDSDDD by capillary electrophoresis [23]. As described above, the phosphorylated product and the unphosphorylated substrate could be separated by their difference in charge. The precisely detectable signal for the phosphorylated product was used to determine the activity of the kinase. After purification, CK2α-pAzF (2.6 µg in 200 µL) exhibited an activity of 3.04 × 10^−5^ µmol/min, which is almost identical to the activity of 3.66 × 10^−5^ µmol/min of the unlabeled purified CK2α (0.2 µg in 200 µL), as reported before by Gratz et al. [21]. This implies that the incorporation of the unnatural amino acid did not substantially alter the kinase activity.

**Figure 6:**
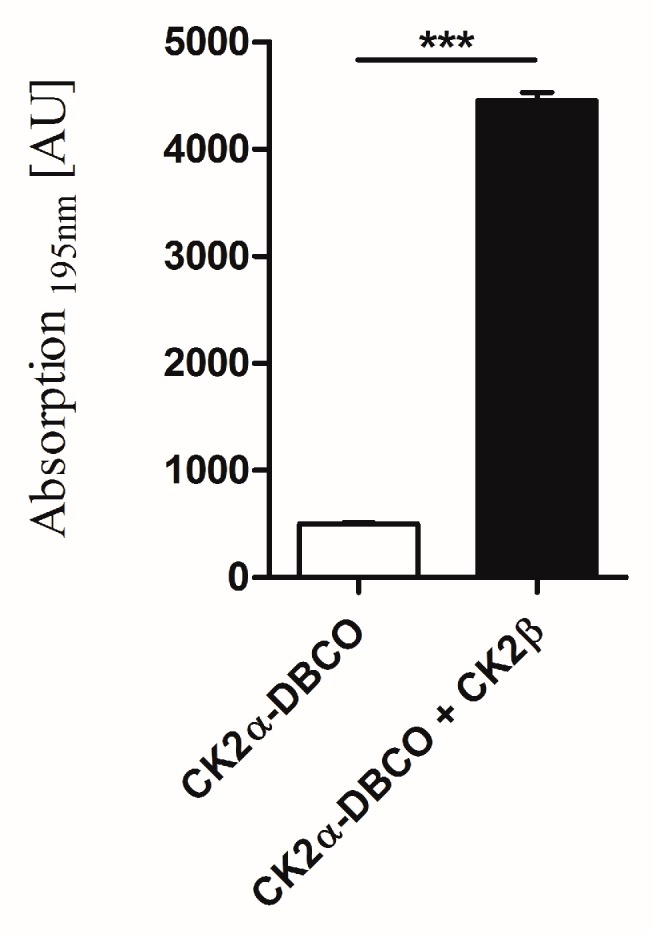
Proof of interaction between CK2β and the CK2α-DBCO-subunit. The activity of CK2α-DBCO545 [**□**] alone and by addition of purified CK2β [**■**] was analyzed by CE assay. There were significant differences in activity (*n* = 3, error bars ± SEM, *** *p* < 0.0001, unpaired *t* test).

In addition, the influence of the unnatural amino acid pAzF followed by the click reaction with DBCO545 on the interaction with the CK2β subunit was investigated. Therefore the phosphorylation activity of the labeled CK2α-DBCO545 alone and in addition of purified CK2β were quantified by capillary electrophoresis (Figure 6). For this purpose, purified regulatory CK2β-subunit was added to CK2α-DBCO545 in a 1:1 ratio. The CK2 holoenzyme, consisting of CK2α-DBCO545 and CK2β, was almost 8 times more active in comparison to the CK2α-DBCO545 subunit alone. This is in accordance with the ratios in the activities of the unlabeled CK2 holoenzyme and CK2α alone as described before [32] and indicates the formation of the CK2 holoenzyme with purified CK2β and CK2α-DBCO545.

**Figure 7:**
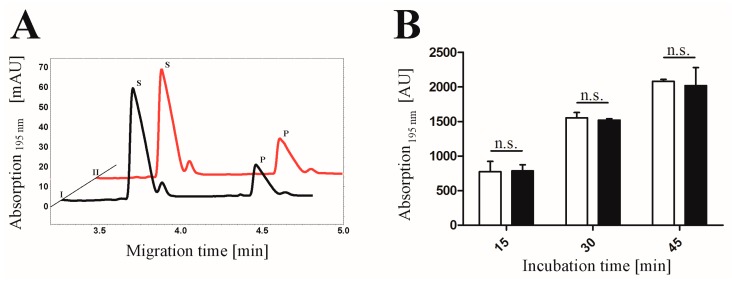
Phosphorylation activity of the heterotetrameric CK2 with or without coupling to DBCO545. (**A**) Comparison of the phosphorylated product between the holoenzyme including CK2α-DBCO545 (I, 2.6 µg) and CK2α-pAzF (II, 2.6 µg) is shown in an electropherogram after 30 min incubation with the substrate RRRDDDSDDD. Substrate (S) and product (P) peaks were detected after 3.7 min and 4.3 min, respectively. (**B**) The activities of the holoenzymes consisting of CK2α-pAzF [**□**] as well as CK2α-DBCO545 [**■**] were analyzed after 15, 30 and 45 min for each sample by CE. Mean values ± standard errors of the means (SEM) from three independent experiments are given (not significant, *p* > 0.05).

In the next step, the activity of the heterotetrameric CK2 (α_2_β_2_) including pAzF before and after the SPAAC click reaction with the fluorophore DBCO545 was tested (Figure 7). The phosphorylated substrate (RRRDDDSDDD) was determined after 15, 30 and 45 min. It turned out as shown in Figure 7B, that there was no significant difference in the phosphorylation activity between the labeled and the non-labeled CK2 holoenzyme (*p* > 0.05). This demonstrates that there was no variation of kinase activity—at least for this peptidic substrate—after performing the SPAAC click reaction with the α-subunit. This indicates for the first time the modification of CK2α with a fluorophore without loss of activity by click chemistry.

### 2.5. Interaction of Surface-Displayed CK2β and CK2α-DBCO545

**Figure 8:**
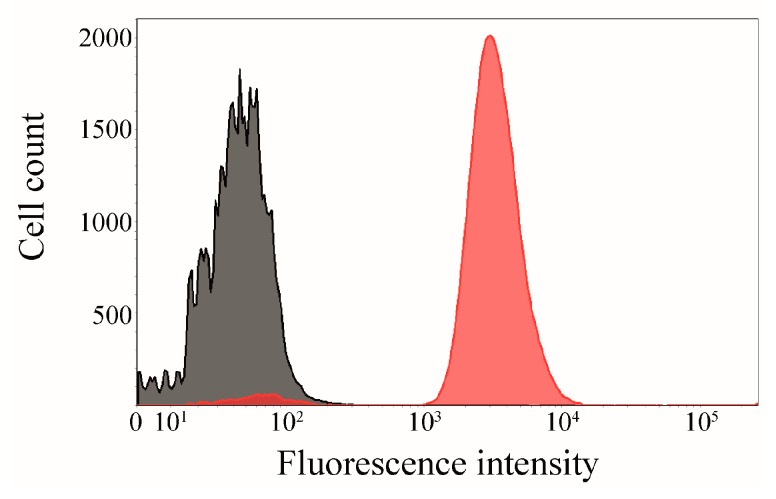
Proof of interaction between surface-displayed CK2β and CK2α-DBCO545-subunit. CK2β, which was translocated on the surface of *E. coli* by Autodisplay, was incubated for 1 h at 37 °C with purified specifically labeled CK2α-DBCO545. The binding affinity of CK2β and CK2α-DBCO545 (red, mF = 3800) was analyzed by flow cytometry. As a non-binding control, surface-displayed sorbitol dehydrogenase [33] was used (grey, mF = 108).

In an additional experiment, *E. coli* BL21(DE3) cells displaying the CK2β subunit (OD_578_ = 1), which was enabled by Autodisplay, were incubated with purified CK2α-DBCO545 for 1 h at 37 °C and subsequently analyzed by flow cytometry (Figure 8). The surface-displayed sorbitol dehydrogenase [33] served as a non-binding control. A higher mean fluorescence intensity (mF) would indicate a specific binding affinity of CK2α-DBCO545. *E. coli* cells displaying CK2β (mF: 3800) showed a significantly higher fluorescence intensity than the control cells displaying sorbitol dehydrogenase (mF: 108). This indicated the specific binding of CK2α-DBCO545 to the surface-displayed CK2β-subunit. The innovatively labeled CK2α in combination with Autodisplay appears to be an advantage for flow cytometry-based screening assays to identify inhibitors of the CK2α/CK2β interaction.

### 2.6. Click Chemistry of CK2β-AT on the Surface of *E. coli*

In addition to the specifically labeled CK2α subunit, Autodisplay [34] was used for a site-directed modification of the CK2β subunit by a fluorophore on the surface of *E. coli*. The subunit CK2β was chosen for the translocation on the surface, because the β-subunit first forms dimers, and subsequently CK2α is bound to yield the formation of heterotetrameric CK2. Tyrosine Y108 of the β-subunit was chosen for the incorporation of the unnatural amino acid pAzF. Besides the structural similarity of tyrosine in comparison to pAzF as described above, the corresponding position is located at the periphery of the β-subunits structure. Y108 has a sufficient distance to the dimerization site of CK2β and to the interaction site with CK2α, which is supposed to facilitate a modification with a fluorophore by click reaction without structural restrictions.

The plasmid pCK2β-AT, which contains the gene encoding the CK2β autotransporter fusion protein (CK2β-AT), previously described by Gratz et al. [21], was used for the incorporation of the unnatural amino acid pAzF followed by the SPAAC click reaction. Consequently, site-directed mutagenesis was used to modify the gene encoding CK2β-AT, resulting in the replacement of the codon for Y108 (TAC) by the amber stop codon TAG. The corresponding plasmid was named pCK2β-AT^Y108Stop^ and encoded a carbenicillin resistance. The biosynthesis of CK2β-AT-pAzF was again controlled by the T7-promotor. *E. coli* BL21(DE3) cells were transformed with both plasmids, pCK2α^Y239Stop^ and pEVOL-pAzF, as described above. The full length CK2β-fusion protein could only be synthesized in case both plasmids were present in one cell and after the addition of both inducers (IPTG/arabinose). Finally, unnatural amino acid pAzF was required to be present in the growth medium as well. In order to analyze the successful incorporation of pAzF in CK2β displayed at the cell surface, the SPAAC click reaction was performed with whole cells of *E. coli*. As a control, bacterial cells displaying CK2β without the incorporated unnatural amino acid on the surface were applied to the same procedure.

**Figure 9:**
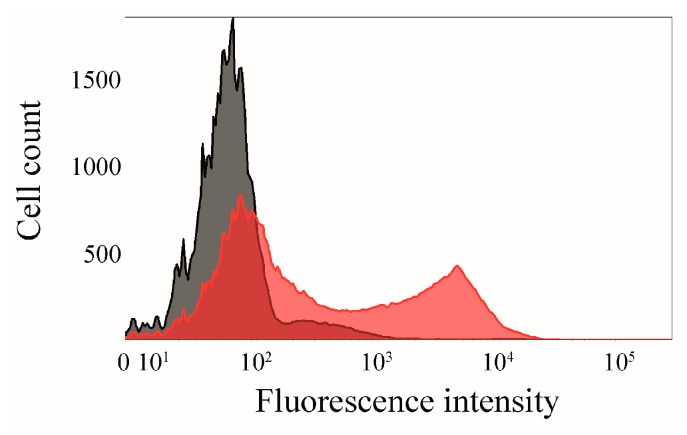
SPAAC reaction of CK2β-AT-pAzF and DBCO545 on the surface of *E. coli*. Cells (OD_578_ = 1) displaying CK2β-AT-pAzF were incubated with the fluorophore DBCO545 (50 µM) for 1h at RT. After three washing steps, *E. coli* displaying CK2β-AT-DBCO545 (red, mF = 1495) were analyzed by flow cytometry. As a control, surface translocated CK2β-AT without incorporated unnatural amino acid pAzF was applied and treated identically (grey, mF = 120).

The density of both cell populations was set to an OD_578_ = 1 and incubated with the fluorophore DBCO545 (50 µM) for 1h at RT. After three washing steps, cells were subsequently analyzed by flow cytometry (Figure 9). It could be shown that there was a significantly higher fluorescence for the *E. coli* cells displaying CK2β-AT-DBCO545 (mF = 1495) in comparison to the control cells (mF = 120). The bioorthogonal click reaction with proteins on the surface of bacterial cells could be used for methods, based on fluorescence detection. Moreover this approach could overcome the need of purifying proteins for binding studies.

### 2.7. Application of CK2α-pAzF for MST Measurements

**Figure 10:**
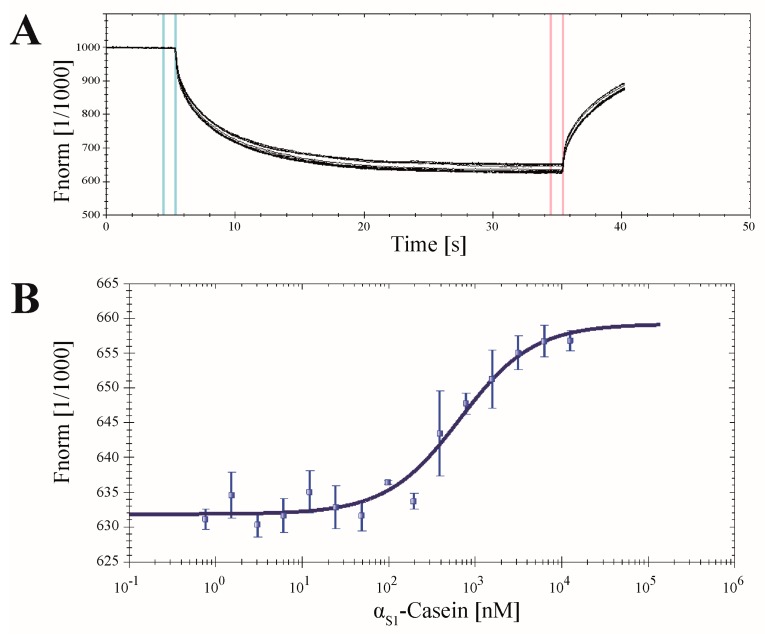
Interaction of CK2α-DBCO-Sulfo-Cy5 and human α_S1_-casein. To a constant amount of CK2α (65 nM) α_S1_-casein was titrated in different concentrations, ranging from 0.76 nM to 12.50 µM. (**A**) The normalized fluorescence signals of the thermophoresis of 15 different dilutions of α_S1_-casein in presence of the CK2α subunit were recorded. (**B**) The *K_D_* value of 631 ± 86.2 nM was determined from three independent experiments using NT Analysis 1.5.41 software (NanoTemper Technologies GmbH, München, Germany).

The specifically labeled CK2α can be used for different approaches based on fluorescence detection. Microscale thermophoresis (MST) represents a relatively new application in the determination of dissociation constants (*K_D_*) of binding partners [13]. Here, the change of thermophoresis of a fluorescent protein induced by the binding of an unlabeled interaction partner is detected. In this study MST measurements were used to determine the dissociation constant of the well-known in vitro CK2 substrate α_S1_-casein [35] with CK2α (Figure 10).

The SPAAC click reaction was performed as mentioned before using the fluorophore DBCO-Sulfo-Cy5. Subsequently, different concentrations of human α_S1_-casein ranging from 0.76 nM to 12.50 µM were added to a constant volume of CK2α-DBCO-Sulfo-Cy5 (65 nM). A difference in thermophoresis of the α-subunit in dependence of the α_S1_-casein concentration was detected and confirmed α_S1_-casein as a binding partner of CK2α (Figure 10A). The difference in the fluorescence levels between the unbound and the bound state resulted in a sigmoidal plot (Figure 10B) and enabled the determination of a *K_D_* value of 631 ± 86.2 nM. This dissociation constant of CK2α and human α_S1_-casein has not been described before. The *K_D_* of CK2 with a bovine casein mixture, however, has been measured by surface plasmon resonance spectroscopy, which was in the same order of magnitude as the *K_D_* for CK2α and human α_S1_-casein as obtained here [36]. This seems to indicate that the CK2α click chemistry in combination with MST measurements provides a convenient method for the identification and characterization of new interaction partners or inhibitors.

## 3. Materials and Method

### 3.1. Bacterial Strain and Culture Conditions

*Escherichia coli* BL21(DE3) was used for the biosynthesis of proteins. Cells were routinely cultivated in lysogeny broth (LB) supplemented with chloramphenicol (30 mg/L) and/or carbenicillin (50 mg/L) depending on the antibiotic-resistance factor(s) encoded on the DNA plasmid(s). For the cultivation of cells with surface-displayed proteins, additionally 10 µM ethylenediaminetetraacetate (EDTA) and 10 mM 2-mercaptoethanol were added to the LB medium. *E. coli*, which were used for the incorporation of the unnatural amino acid pAzF, were routinely grown in minimal medium (pH 7, 34 mM Na_2_HPO_4_, 22 mM KH_2_PO_4_, 100 µM CaCl_2_, 1 mM MgSO_4_, 30 µg/mL thiamine, 0.1% NH_4_Cl, 0.2% glucose, 22 nM Fe(III)Cl_3_). Bacterial cells were grown at 37 °C with shaking (200 rpm) until an OD_578_ of 0.6 was reached. Protein expression was induced by the addition of isopropyl-β-d-thiogalactopyranoside (IPTG) to a final concentration of 1 mM and/or arabinose (0.2%).

### 3.2. Design of pCK2α^Y239Stop^ and pCK2β-AT^Y108Stop^ Plasmids

For the generation of the mutant pCK2α^Y239Stop^, the plasmid pT7-7CK2α was used as template. Site-directed mutagenesis was performed by the use of the QuickChange protocol (Stratagene) and the following primers, where the mutated codon is shown in boldface: 5′-CCTCACCAACTGATC**CTA**ATTGTCATGTCCATG-3′ and 5′-CATGGACATGACAAT**TAG**GATCAGTTGGTGAGG-3′. Plasmid pCK2α^Y239Stop^ was obtained. The design and construction of the plasmid encoding for the autotransporter fusion protein pCK2β-AT has already been described in detail earlier [21]. For the construction of pCK2β-AT^Y108Stop^, the plasmid pCK2β-AT was used as template. Site-directed mutagenesis by the use of the primers (mutation in boldface), 5′-GTACACACGAGGACA**CTA**ACCAAAGTCTCCTTG-3′ and 5′-CAAGGAGACTTTGGT**TAG**TGTCCTCGTGTGTAC-3′, resulted in pCK2β-AT^Y108Stop^. Both obtained DNA plasmids were verified by DNA-sequencing (Seqlab, Göttingen, Germany).

The plasmid pEVOL-pAzF [25] encoding for the amber suppressor tRNA/aminoacyl-tRNA synthetase was a gift from Peter Schultz (Addgene plasmid #31186).

### 3.3. Biosynthesis and Purification of CK2α-pAzF

For the recombinant expression of the mutant CK2α-pAzF, plasmids pCK2α^Y239Stop^ and pEVOL-pAzF [25] were used to transform the bacterial strain *E. coli* BL21(DE3). An overnight culture (LB medium) was inoculated with a single colony. For protein biosynthesis, 12 mL overnight culture was added to 1200 mL minimal medium (3.1). Cells were grown in minimal medium with 30 mg/L chloramphenicol and 50 mg/L carbenicillin at 37 °C (200 rpm) until an OD_578_ of 0.6 was reached. The volume of the cell suspension was reduced to 120 mL, the unnatural amino acid pAzF (Bachem AG, Bubendorf, Switzerland) was supplemented in a final concentration of 1 mM and dissolved by shaking (200 rpm) for 15 min at 37 °C. Biosynthesis of protein CK2α-pAzF was induced by addition of IPTG to a final concentration of 1 mM. Arabinose was added (0.2%) for the synthesis of the orthogonal aminoacyl-tRNA synthetase. After 4 h at 30 °C (200 rpm) bacteria were harvested (3000× *g*, 10 min, 4 °C) and stored at −80 °C. Protein purification was done by a modified protocol of Grankowski et al. [30]. Cells were resuspended in 30 mL buffer P300 (25 mM Tris/HCl (pH 8.5), 300 mM NaCl, 7 mM 2-mercaptoethanol) supplemented with 0.2 mM PMSF, 0.5 mg/L leupeptin and 0.7 mg/L pepstatin. Bacterial cells were disrupted by sonication (six 20 s cycles, with 20 s intervals on ice) and centrifuged (100,000× *g*, 120 min, 4 °C). The obtained supernatant was used for the purification of CK2α-pAzF and loaded on a P11 phosphocellulose column, equilibrated with P300. A linear gradient of 600 mL P300–P1500 (25 mM Tris/HCl (pH 8.5), 1500 mM NaCl, 7 mM 2-mercaptoethanol) was applied. The protein kinase eluted at a NaCl concentration of approximately 600–700 mM, which was analyzed by SDS-PAGE. After dialysis against buffer P50 (25 mM Tris/HCl (pH 8.5), 50 mM NaCl), the protein was stored at −80 °C. For SDS-PAGE, the CK2α-pAzF was concentrated by ultrafiltration using vivaspin500 columns (Sartorius, Göttingen, Germany). The protein concentrations were determined in triplicate by NanoPhotometer Pearl (Implen, München, Germany).

### 3.4. Surface Display of CK2β-AT-pAzF

For the surface display of the mutant CK2β-AT-pAzF, plasmids pCK2β-AT^Y108Stop^ and pEVOL-pAzF [25] were used to transform the bacterial strain *E. coli* BL21(DE3). An overnight culture (LB medium) was inoculated with a single colony. For the biosynthesis of CK2β-AT-pAzF, 1 mL overnight culture was supplemented to 40 mL minimal medium (3.1.) containing 30 mg/L chloramphenicol and 50 mg/L carbenicillin. Cells were grown at 37 °C (200 rpm) until an OD_578_ of 0.6 was reached and the unnatural amino acid pAzF was added (1 mM). After dissolving pAzF by shaking (200 rpm) for 15 min at 37 °C, the translocation of CK2β-AT-pAzF to the surface of *E. coli* was induced by addition of IPTG (1 mM). For the synthesis of the orthogonal aminoacyl-tRNA synthetase, arabinose was added (0.2%). After an incubation for 2 h at 30 °C, bacteria were harvested (3000× *g*, 10 min, 4 °C) and stored in kinase buffer (3.7.) at 4 °C.

### 3.5. SDS-PAGE

Protein samples were diluted 1:1 with SDS sample buffer (100 µM Tris/HCl (pH 6.8), 4% SDS, 0.2% bromophenol blue and 20% glycerol, 200 mM DTT), boiled for 20 min at 95 °C and loaded onto a SDS-Gel containing 10% acrylamide. PAGE Ruler prestained protein marker (Fermentas, St. Leon-Roth, Germany) was used as a molecular weight standard. After separation, protein bands were stained with Coomassie brilliant blue G250 (Serva, Heidelberg, Germany). Proteins linked to the fluorescent dye DBCO545, were visualized by a LED-illuminator Gel Jet Imager (Intas, Göttingen, Germany).

### 3.6. SPAAC Reaction of CK2α-pAzF

The purified protein kinase CK2α-pAzF (130 µg/mL) in buffer P50 was treated with DBCO545 (Jena Bioscience, Jena, Germany) or DBCO-Sulfo-Cy5 (Jena Bioscience, Jena, Germany) in a final concentration of 50 µM. After 1 h in the dark at room temperature (RT) the reaction solution with the obtained CK2α-DBCO-fluorophore was directly applied for SDS-PAGE, CE-measurements or flow cytometry. In case of MST measurements an additional ultrafiltration step using vivaspin500 columns (Sartorius, Göttingen, Germany) was established in order to remove the unbound fluorophore.

For the SPAAC reaction of CK2β-AT-pAzF on the surface of *E. coli*, cell density was set to OD_578_ = 1 and the click reaction was performed with DBCO545 (50 µM) for 1 h in the dark at RT. Cells were washed three times with PBS to remove unbound DBCO545 and subsequently used for flow cytometry measurement.

### 3.7. CE-based Activity Measurements of CK2

The determination of the activity of the mutated human protein kinase CK2α-pAzF was based on capillary electrophoresis (CE) assay [23]. In this work, 20 µL CK2α-pAzF alone or in addition of 10 µL CK2β in kinase buffer (50 mM Tris/HCl (pH 8.5), 25 mM NaCl, 20 mM MgCl_2_ and 1 mM DTT) were incubated at 30 °C for 10 min. CK2β was purified as described in Grankowski et al. [30]. The phosphorylation reactions were started by adding 120 µL of assay buffer (25 mM Tris/HCl (pH 7.5), 50 mM NaCl, 20 mM MgCl_2_, 1 mM DTT, 190 µM substrate peptide RRRDDDSDDD (Genic Bio, Shanghai, China) and 100 mM ATP). The final concentration in the total reaction volume of 200 µL were 114 µM substrate peptide and 600 µM of ATP. After different incubation times the enzyme activity was stopped by transferring the samples to a 96 well-microplate where EDTA (12.5 mM final concentration) was supplemented to eliminate any free divalent cations and by reducing the temperature to 4 °C. The phosphorylation of the substrate peptide was analyzed by a Beckman Coulter pa800 plus (Krefeld, Germany) CE system.

Fluorescein-5-isothiocyanate (FITC) labeling of CK2 was performed using a kit from Calbiochem (Merck, USA) according to manufacturer´s protocol. CK2-FITC was applied in the same concentration as CK2α-pAzF and treated identically in the CE-based assay.

### 3.8. Flow Cytometry

*E. coli* cells genetically prepared to display the fusion protein CK2β-AT [21] were grown to the mid log phase (OD_578_ = 0.6), harvested and washed three times with kinase buffer. Cell density was adjusted to OD_578_ = 1. 20 µL bacterial cells and 10 µL CK2α-DBCO545 were mixed and incubated for 60 min at 37 °C. Afterwards the mixture was washed three times with sterile filtered PBS buffer to remove unbound CK2α-DBCO545 and stored on ice. *E. coli* cells displaying CK2β-AT-pAzF were grown (3.4.), density was set to OD_578_ = 1 and the click reaction was performed with DBCO545 (50 µM). After three washing steps with sterile filtered PBS buffer to remove unbound DBCO545, cells were stored on ice. Flow cytometry measurements were performed with a FACS Aria III (BD, Heidelberg, Germany) using a 561 nm laser for excitation and 610/20 BP- and 600 LP-filter for detection, respectively. For each sample, 50,000 cells were recorded and analyzed by FACSDIVA 8.0 software (BD, Heidelberg, Germany).

### 3.9. Microscale Thermophoresis (MST)

For the determination of the *K_D_* value of CK2α and α_S1_-casein the Monolith NT.115 (NanoTemper Technologies GmbH, München, Germany) was used. Protein α_S1_-casein was purified as described by Vordenbäumen et al. [35]. First the CK2α-pAzF was coupled to DBCO-Sulfo-Cy5 via SPAAC click reaction (3.5). The concentration of the labeled protein was obtained by linear regression of different fluorophore concentrations and subsequent detection by the Monolith NT.115. Afterwards 10 µL of 65 nM specifically labeled CK2α in kinase buffer (50 mM Tris/HCl (pH 8.5), 25 mM NaCl, 20 mM MgCl_2_) including 0.1% Tween-20 were mixed with 10 µL α_S1_-casein (20 mM HEPES (pH7.2)) in different concentrations ranging from 1.5 nM to 25 µM. Each sample was incubated at 37 °C for 90 min to assure the complete renaturation of α_S1_-casein [37]. Fluorescence (red filter, LED power 50%) and thermophoresis (MST power 60%) were recorded at 30 °C for 30s. The *K_D_* value was determined from three independent experiments using NT Analysis 1.5.41 software (NanoTemper Technologies GmbH, München, Germany).

## 4. Conclusions

The innovative modification of the subunit CK2α-pAzF with a DBCO-fluorophore offers various advantages for the protein kinase CK2 in contrast to commercially available labeling reagents like FITC. In combination with Autodisplay, the application of CK2α-pAzF could be a significant advancement for screening assays by flow cytometry and for CK2α/CK2β interaction studies. The successful modification of surface-displayed CK2β-AT by the fluorophore DBCO545 was established, which could overcome the need for protein purification of CK2β and could enable measurements with whole cells. CK2α-pAzF also facilitated screening for new inhibitors by MST measurements. The determined *K_D_* value for the CK2α binding to α_S1_-casein indicated the possibility for the characterization of further interaction partners of the cancer target CK2. Besides modifying CK2 with fluorophores, modification of CK2 by click chemistry could be an advantage in the development of screening assays for binding partners and inhibitors, unlocking the potential of site-directed immobilization, i.e., preferable for ELISA or surface plasmon resonance spectroscopy (SPR).
